# Species, sex and geo-location identification of seized tiger (*Panthera tigris tigris*) parts in Nepal—A molecular forensic approach

**DOI:** 10.1371/journal.pone.0201639

**Published:** 2018-08-23

**Authors:** Dibesh Karmacharya, Adarsh M. Sherchan, Santosh Dulal, Prajwol Manandhar, Sulochana Manandhar, Jyoti Joshi, Susmita Bhattarai, Tarka R. Bhatta, Nagendra Awasthi, Ajay N. Sharma, Manisha Bista, Nawa R. Silwal, Pravin Pokharel, Rom R. Lamichhane, Netra Sharma, Bronwyn Llewellyn, Claudia Wultsch, Marcella J. Kelly, Digpal Gour, Lisette Waits, Jean-Marc Hero, Jane Hughes

**Affiliations:** 1 Center for Molecular Dynamics Nepal, Thapathali-11, Kathmandu, Nepal; 2 School of Environment, Griffith University, Gold Coast, Queensland, Australia; 3 Central Investigation Bureau (CIB), Pillar 4, Nepal Police, Kathmandu, Nepal; 4 Bio-Diversity Section, Ministry of Forest and Soil Conservation, Kathmandu, Nepal; 5 Environment Team, U.S. Agency for International Development (USAID), Kathmandu, Nepal; 6 Sackler Institute for Comparative Genomics, American Natural History Museum, New York, New York, United States of America; 7 Department of Fish and Wildlife Conservation, Virginia Tech, Blacksburg, Virginia, United States of America; 8 Laboratory for Ecological, Evolutionary and Conservation Genetics, University of Idaho, Moscow, Idaho, United States of America; 9 School of Science and Engineering, University of the Sunshine Coast, Sunshine Coast, Queensland, Australia; 10 Durrell Institute of Conservation and Ecology, School of Anthropology and Conservation, University of Kent, Canterbury, United Kingdom; Smithsonian Conservation Biology Institute, UNITED STATES

## Abstract

Tiger (*Panthera tigris*) populations are in danger across their entire range due to habitat loss, poaching and the demand for tiger parts. The Bengal tiger (*Panthera tigris tigris*) is an endangered apex predator with a population size estimated to be less than 200 in Nepal. In spite of strict wildlife protection laws, illegal trade of tiger parts is increasing; and Nepal has become one of the major sources and transit routes for poached wildlife parts. Identification of wildlife parts is often challenging for law enforcement officials due to inadequate training and lack of available tools. Here, we describe a molecular forensic approach to gain insight into illegally trafficked tiger parts seized across Nepal. We created Nepal’s first comprehensive reference genetic database of wild tigers through the Nepal Tiger Genome Project (2011–2013). This database has nuclear DNA microsatellite genotype and sex profiles, including geo-spatial information, of over 60% (*n* = 120) of the wild tigers of Nepal. We analyzed 15 putative cases of confiscated poached tiger parts and all were confirmed to be of tiger. Ten samples were identified as male and five were female. We determined probable geo-source location for 9 of the 14 samples with 6–8 nuclear DNA microsatellite loci using inferences from four different statistical assignment methods. Six samples were assigned to Bardia National Park and one of these was an exact match to a female tiger previously profiled in our fecal DNA reference database. Two tiger samples were assigned to Shuklaphanta Wildlife Reserve and one to Chitwan National Park. We are unable to definitively assign five tiger samples which could be offspring dispersers or might have come from tiger population outside of Nepal. Our study revealed that the western region, particularly Bardia National Park, is a poaching hotspot for illegal tiger trade in Nepal. We present feasibility of using molecular forensic based evidence to incriminate criminals in a court of law in the fight against wildlife crime.

## Introduction

The Bengal tiger (*Panthera tigris tigris*) is the most prevalent and endangered tiger subspecies found in the Indian subcontinent [1, 2]. In just over a century, wild tiger populations have dramatically fallen (>97%) and currently about 3,200 tigers are left in the wild [1, 2]. Widespread deforestation, habitat fragmentation and loss, prey depletion, and poaching are the major causes of tiger population decline [3–6]. Moreover, diseases such as canine distemper virus (CDV) could pose further threat to already declining tiger populations [7]. Poaching and illegal wildlife trade present the greatest threats to the survival of tigers across their range [8–10]. With increasing demand for wildlife parts and illicit trade of wildlife, Asia has become one of the major hubs for wildlife crimes [4, 11, 12]. Nepal is one of the major sources of illegal wildlife parts [13, 14]. Each year lucrative, illicit wildlife commodities including tiger parts such as skin, bone, claws, teeth, blood, genitals, and meat are illegally trafficked to Chinese markets [14, 15] and used in traditional Chinese medicine [15–17]. Wildlife crime in Nepal is driven by a multitude of factors, including poverty, lucrative financial rewards from illicit wildlife products in black markets, porous borders, lack of conservation awareness, and poor law enforcement for the prevention of wildlife crimes [18, 19].

Often the only evidence recovered from wildlife crime scene is traces of blood, pieces of meat, skin, fur, bones and other forms of biological materials. Many of the seized specimens are virtually impossible to identify based on morphological analysis alone. Lack of precise identification of confiscated specimens has often resulted in no convictions in court of law in Nepal. Hence, there is an urgent need to use advanced forensic techniques to identify seized wildlife specimens to determine their species and region of origin.

Molecular forensics can provide information on species, sex and individuals of poached animals with relatively high accuracy [20–29]. Using a geo-spatial reference genetic database, it is possible to determine the source of the seized parts using various statistical and computational analyses.

Applications of DNA profiling in wildlife forensics were successfully applied for whales and dolphins [30, 31]; mule deer, white-tailed deer, moose, caribou and American black bear [32]; wild boar and wolves [33, 34]; African elephants [20, 23, 35] and leopards [24]. For tigers, molecular forensic techniques have been, for example, used to investigate a tiger bone smuggling case in China [36] and illegal sale of tiger meat from a circus tiger [37]. In Nepal, utility of genetic tools in both population studies and forensics have recently been explored for tigers, through the Nepal Tiger Genome Project (NTGP) (2011–2013) [38].

Genetic tools combined with geo-location baseline data on species can discern geographic origin of confiscated wildlife parts and their derivatives [20, 23, 24, 29, 32, 35, 39]. This kind of information is highly valuable for conservation managers to prioritize their efforts in effective anti-poaching activities. Best practices in anti-poaching efforts require scientifically sound wildlife monitoring efforts and the effective sharing of information amongst conservation managers and relevant stakeholders [40]. The Government of Nepal (GoN) has asserted that the incidences of seized tiger parts have dramatically increased over the last years. Nepal is both a source and a conduit for wildlife parts trafficking into China/Tibet and other areas. Hence there is a need to utilize effective tools such as molecular forensics to track poaching and illegal trade in tiger parts within the country and beyond. The GoN has proposed establishing a system to better track records of poaching incidences, confiscation and enforcement of the Convention of International Trade in Endangered Species (CITES) of Wild Fauna and Flora by increasing the in-country capacity for identification of animal parts and their derivatives using effective and modern DNA-based forensic tools [41].

The primary goal of this study is to apply a molecular forensic approach to identify species, sex and the population of origin of 15 putative tiger parts confiscated by the Central Investigation Bureau (CIB) of Nepal Police. To identify the geo-location source of these samples, we used inferences from four different assignment methods and a previously developed geo-spatial genetic reference database of wild tigers of Nepal [38]. The database includes genetic and geo-location information on 120 wild tigers of Nepal, covering 60% of the estimated 200 tigers across the Terai Arc Landscape (TAL) sampled across the main tiger habitats (Banke National Park [BaNP], Bardia National Park [BNP], Chitwan National Park [CNP], Parsa Wildlife Reserve [PWR] and Suklaphanta Wildlife Reserve [SWR]) [42, 43].

## Materials and methods

### Source of forensic samples

The laboratory of the Center for Molecular Dynamics Nepal (CMDN) received a total of fifteen seized putative tiger parts, which included thirteen skin pieces, and two blood smeared knives (S1 Fig) seized by the CIB during investigative operations (2014–16) in southern Nepal (S1 Table). These samples were labeled, photographed and stored with desiccant at room temperature. This study has been authorized through study permit provided by the Department of National Parks and Wildlife Conservation, Ministry of Forests and Soil Conservation-Government of Nepal.

### Geo-spatial baseline genetic database of wild tiger in Nepal

**Fig 1:**
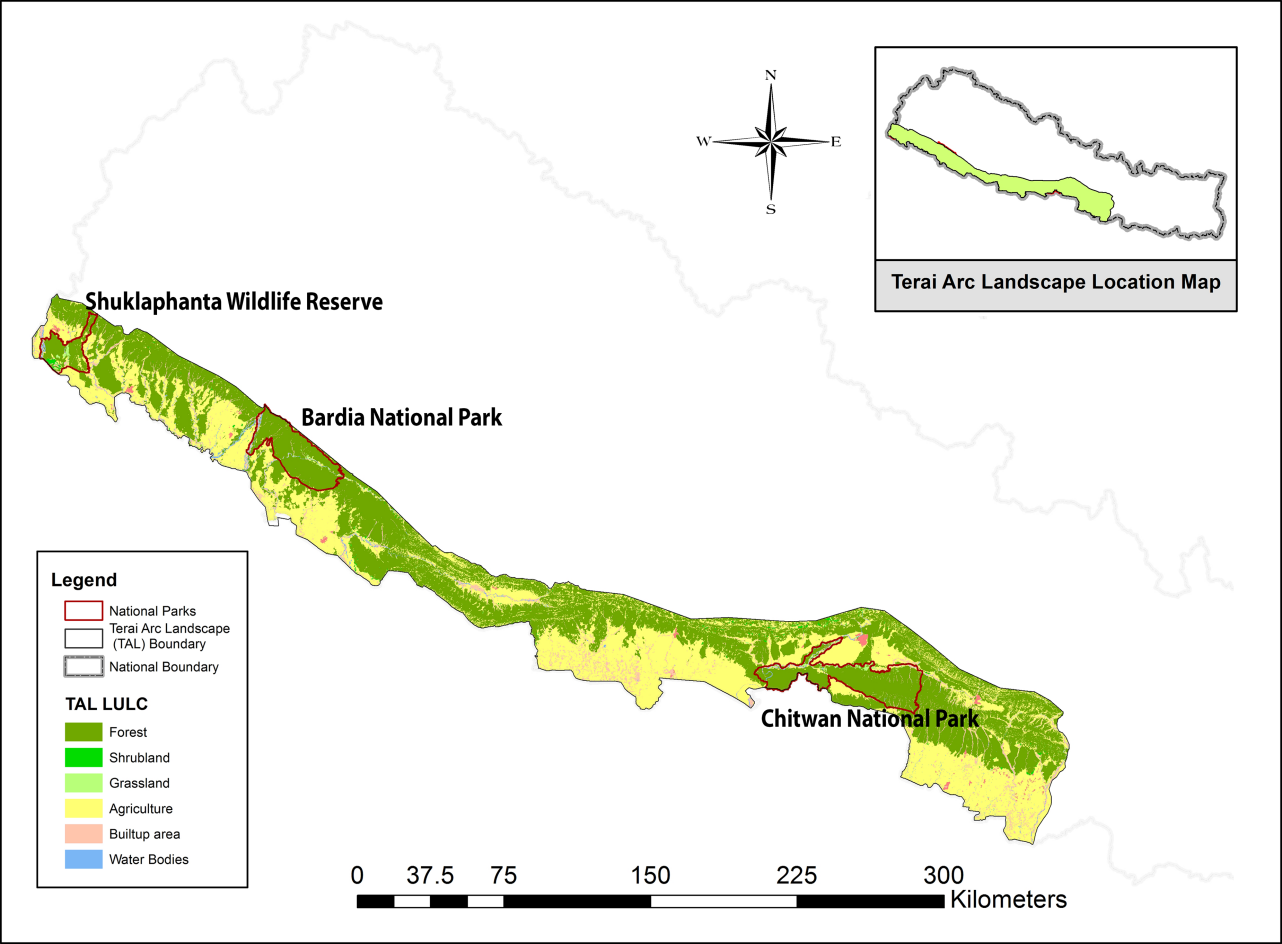
Scat sample collection (*n* = 770; CNP = 420, BNP = 116, SWR = 79, PWR and wildlife corridors = 155) sites for tiger baseline genetic database under Nepal Tiger Genome Project (NTGP, 2011–2013). The estimated tiger population of Nepal was 198 (CNP = 120, BNP = 50, SWR = 17, PWR = 7, BaNP = 4)[45].

NTGP created Nepal’s first comprehensive tiger reference genetic database by collecting and analyzing fecal samples (*n* = 770;CNP = 420, BNP = 116, SWR = 79, PWR and wildlife corridors = 155) across the TAL region of Nepal (Fig 1 and S2 Table). This database includes species, sex and individual DNA microsatellite profiles of 120 wild tigers[44].

### Genetic analyses of forensic samples

#### DNA extraction

DNA was extracted using DNeasy Blood and Tissue Kit following the manufacturer’s instruction (Qiagen Inc., Germany)[46]. A 1 cm^2^ piece of skin (forensic) was digested overnight at 56°C in 180 μL ATL and 20 μL Proteinase K solution. Extracted DNA was precipitated with ethanol and purification was conducted in a spin column following the kit’s protocol. For the blood smeared knives, samples were taken by swabbing the smeared surface with a sterile cotton swab saturated with phosphate buffer saline. DNA from swab samples were extracted following the same protocol used in skin samples. Each batch of DNA extractions included a negative extraction control. Precaution was taken to avoid contamination. Extracted DNA was stored at -20°C.

#### Species identification

Tigers were identified using a PCR assay that used tiger specific mtDNA Cytochrome-b (*CYT-B*) primers[47]. A total of 7 μL PCR reaction was prepared containing 3.5 μL of 2X Qiagen multiplex mastermix (Qiagen Inc., Germany), 0.7 μL Q-solution, 200 nM each *CYT-B* primer sets and 1.5 μL of purified DNA template. The thermocycling condition was 95°C for 15 min followed by 35 cycles of 94°C for 30 sec, 59°C for 90 sec and 72°C for 90 sec with the final extension at 72°C for 10 min. Amplified 162 bp target PCR product was visualized under 1.5% agarose gel electrophoresis (S2 Fig).

#### Sex identification

Sex of identified tiger samples was determined by amplifying the Amelogenin (*AMEL*) gene of sex chromosomes[48]. A 7 μL PCR reaction was prepared containing 3.5 μL of 2X Qiagen multiplex mastermix (Qiagen Inc., Germany), 0.7 μL Q-solution, 200 nM each *AMEL* forward and reverse primers and 1.5 μL of purified DNA template. The thermocycling condition was 95°C for 15 min followed by 45 total cycles of 94°C for 30 sec, 53°C for 60 sec and 72°C for 60 sec with final extension at 72°C for 10 min. Amplified PCR products were visualized in 3% agarose gel electrophoresis. Female samples yielded a single (214 bp) PCR band, while two bands (194 bp and 214 bp) identified male samples. Each sample was run in triplicate. Samples that had amplification of X and Y alleles on at least two out of three replicates were identified as male. Samples that had only X allele amplification in all three replicates were assigned as female [49].

#### Individual identification

Individual tigers were identified using a panel of polymorphic microsatellite markers (*n* = 10) developed from the domestic cat (*Feliscatus*) and tiger genomes[50–52]. The primers are arranged in a single multiplex panel. PCR amplification was carried out in a 7 μL reaction volume containing 3.5 μL of Qiagen multiplex master-mix (Qiagen Inc., Germany), 0.7 μL of Q solution, each ten microsatellite primers (**FCA391**: 0.2 μM, **PttD5**: 0.07 μM, **FCA232**: 0.14 μM, **FCA304**: 0.07 μM, **F85**: 0.30 μM, **FCA441**: 0.14 μM, **FCA043**: 0.09 μM, **F53**: 0.49 μM, **FCA205**: 0.21 μM and **PttA2**: 0.07 μM) and 2.5 μL of purified DNA template. The PCR thermocycling condition was 95°C for 15 min with a 10 cycles of touchdown step (94°C for 30 sec, initial annealing at 62°C reduced by 0.5°C in each cycle for 90 sec and extension at 72°C for 60 sec), followed by 25 cycles of 94°C for 30 sec, 57°C for 90 sec and 72°C for 60 sec, and a final extension at 72°C for 10 min. Amplified product (0.7 μL) was genotyped by adding 0.3 μL of LIZ-500 size standard in ABI 310 genetic analyzer (Applied Biosystems, USA). Microsatellite alleles were scored using GENEMAPPER, version 4.1 (Applied Biosystems, USA). To finalize the consensus genotypes, a multi-tube approach was used where at least three identical homozygote PCR results were required for a homozygote genotype call; each allele was observed in two independent PCRs to record a heterozygous genotype [53]. By utilizing the matching tool in GenAlEx version 6.503 [54], individual tigers were identified from genotype data.

#### Genetic structure analysis

The 8 loci microsatellite genotypes from previously identified tiger individuals were used as the reference baseline data [38]. A Bayesian clustering approach was applied to determine the genetic structure in tiger population of Nepal using STRUCTURE, version 2.3.4 [55, 56]. The Bayesian clustering was implemented using 2,000,000 Markov chain Monte Carlo (MCMC) replications after initial 500,000 burn-in with repetitions of the analysis for 10 independent times per each assumed population (*K* = 1 to 6). This analysis was run using the admixture model with correlated allele frequencies and was tested under supervised (with LOCPRIOR) and unsupervised (without LOCPRIOR) learning algorithms. In the supervised method, sampling location information consisting of three geographical locations (CNP, BNP, SWR) were provided as *a priori* information, whereas it was not provided in unsupervised method. The rate of change of likelihood (delta *K*) value was estimated by Evanno method [57] to determine the optimal numbers of genetic clusters or populations present in our reference genotype data for both supervised and unsupervised analysis using STRUCTURE HARVESTER, version 0.6.94 [58]. The percentage of inferred ancestry (*Q*-scores) for each sample from 10 independent runs were aggregated into an average *Q*-scores using CLUMPP, version 1.1.2 [59].

#### Geo-source assignment of unknown tiger samples

Confiscated tiger samples were assigned to potential source populations (reference baseline data obtained through NTGP) using four different approaches, including Bayesian clustering analysis with STRUCTURE, version 2.3.4 [55, 56], likelihood and Bayesian-based assignment in Geneclass, version 2 [60], and multivariate discriminant analysis of principal components (DAPC) using the R package *adegenet*, version 2.1.1 [61]. First, STRUCTURE analysis using the same parameters as described above was performed by combining the genotype data from the forensic samples (unknown origin; PopInfo = 0) with the tiger samples from NTGP’s reference database (known origin; PopInfo = 1). The assignment of unknown samples to sub-populations was determined based on *Q*-score membership values. Tigers were identified as residents of an area when their average *Q* value score was ≥ 70%. We classified individuals with *Q* scores < 70% as admixed and unassigned [62]. Next, we estimated the probability of assignment for each forensic tiger sample to different source populations with assignment threshold score of 0.05 using Paetkau’s frequency based method [63] and with a missing allele frequency of 0.01 using Rannala and Mountain’s Bayesian method [64] in Geneclass, version 2 [60]. We assigned a tiger sample to a population of origin when the probability of assignment was >0.99 and classified samples as unassigned when the probability was below this threshold. To complement these analyses, we also conducted a discriminant analysis of principal components (DAPC) using the R package *adegenet*, version 2.1.1 [61], to cluster genetically similar individuals based on their multi-locus genotype. To establish conservative criteria for forensic assignment, we required a sample to meet the assignment criteria defined above for three of the four methods. These four methods were chosen because they have been shown to perform well in accurately assigning individuals in multiple studies [29, 65, 66].

## Results

### Genetic analyses

A total of 120 individual tigers (CNP = 64; BNP = 36; SWR = 20) were identified by matching tool implemented via GenAlEx version 6.503[54]. We calculated probability of identification (P_ID_) to be 1.5E-06 and probability of identity among siblings (P_ID(sibs)_) to be 3.2E-03 (<0.01) for 8 loci and achieved an overall genotyping success of 40%. Of all the evaluated microsatellite markers, eight were selected for genetic analysis of all samples with fairly high overall PCR amplification success (84%) and genotyping accuracy (82%) (S3 Table). Two microsatellite markers (FCA205 and PttA2) were included in the PCR multiplex but were not considered in the individual identification as one (Locus FCA205) did not amplify in the majority of samples and the other (PttA2) was not polymorphic. Similarly, the mean allelic dropout rate was 2.46% and false alleles were around 15.85% [44].

All forensic samples (*n* = 15) were of tiger (S2 Fig). Sex identification revealed ten males and five females (S3 Fig). Genotyping success was 100% for all forensic samples (S4 Table), except for one of the blood-smeared knife samples (F-NP-0012) in which only half the number of loci successfully amplified. A multiple genotype (microsatellite loci) pattern was observed in F-NP-0012, where three different alleles were detected at three loci *FCA391*, *FCA232* and *FCA043* hence, this sample was excluded from further analyses. By using matching tool in GenAlEx, version 6.503[54], the genotype of one of the forensic samples (F-NP-0004), determined to be originating from a female tiger [Table 1]), matched 100% to previously profiled female tiger at the BNP site in the NTGP baseline tiger reference database.

**Table 1:** Species, sex and location assignment of all forensic samples. Species and sex profiles of 15 forensic samples. *Q*-score values from STRUCTURE, frequency assignment likelihood and Bayesian assignment likelihood values from Geneclass2, posterior membership probabilities from DAPC analyses of 14 forensic samples.

Sample ID	Genetic profile		STRUCTURE *Q* values			GeneClass2 Assignment Likelihoods			Geneclass 2 Bayesian Assignment Likelihoods			DAPC			Forensic Assignment	Probable origin
	Species	Sex	BNP	SWR	CNP	BNP	SWR	CNP	BNP	SWR	CNP	BNP	SWR	CNP		
F-NP-0001	Tiger	Male	0.287	0.629	0.084	0.376	0.611	0.013	0.252	0.747	0.001	**0.998**	0.002	0.000	Unassigned—Admixed BNP/SWR	
F-NP-0002	Tiger	Male	0.265	**0.721**	0.015	0.000	**0.999**	0.000	0.000	**0.999**	0.000	0.000	**1.000**	0.000	SWR	Western Terai
F-NP-0003	Tiger	Male	0.461	0.500	0.039	0.669	0.220	0.110	**0.807**	0.073	0.119	**1.000**	0.000	0.000	Unassigned—Admixed BNP/SWR/CNP	
F-NP-0004	Tiger	Female	**0.817**	0.159	0.023	**0.998**	0.002	0.000	**0.999**	0.000	0.000	**0.999**	0.001	0.000	BNP	Western Terai
F-NP-0005	Tiger	Female	0.535	0.450	0.016	0.707	0.293	0.000	0.709	0.290	0.000	**0.987**	0.013	0.000	Unassigned—Admixed BNP/SWR	
F-NP-0006	Tiger	Female	**0.713**	0.261	0.026	**0.991**	0.008	0.000	**0.997**	0.003	0.000	**0.995**	0.005	0.000	BNP	Western Terai
F-NP-0007	Tiger	Male	0.394	0.577	0.029	0.626	0.350	0.024	0.831	0.137	0.003	**1.000**	0.000	0.000	Unassigned—Admixed BNP/SWR	
F-NP-0008	Tiger	Female	0.540	0.127	0.334	0.865	0.000	0.135	0.641	0.000	0.359	**1.000**	0.000	0.000	Unassigned—Admixed BNP/CNP	
F-NP-0009	Tiger	Male	0.089	0.178	**0.733**	0.000	0.000	**0.999**	0.000	0.000	**0.999**	0.002	0.000	**0.998**	CNP	Eastern Terai
F-NP-0010	Tiger	Male	**0.815**	0.166	0.020	**0.991**	0.009	0.000	**0.984**	0.016	0.000	**0.999**	0.001	0.000	BNP	Western Terai
F-NP-0011	Tiger	Male	**0.916**	0.066	0.018	**0.999**	0.000	0.000	**0.999**	0.000	0.000	**1.000**	0.000	0.000	BNP	Western Terai
F-NP-0013	Tiger	Male	**0.884**	0.095	0.021	**0.999**	0.000	0.000	**0.999**	0.000	0.000	**1.000**	0.000	0.000	BNP	Western Terai
F-NP-0014	Tiger	Male	0.225	**0.756**	0.019	0.000	**0.999**	0.000	0.001	**0.999**	0.000	**0.919**	0.081	0.000	SWR	Western Terai
F-NP-0015	Tiger	Female	0.669	0.233	0.098	**0.999**	0.000	0.000	**0.999**	0.000	0.000	**1.000**	0.000	0.000	BNP	Western Terai
F-NP-0012	Tiger	Male	-	-	-	**-**	-	-	**-**	-	-	-	-	-	Genotyping failed	-

Bold highlighting indicates that a sample meets defined criteria for assignment ≥70% for STRUCTUREand≥99% for Geneclass2

### Reference population genetic structure

**Fig 2:**
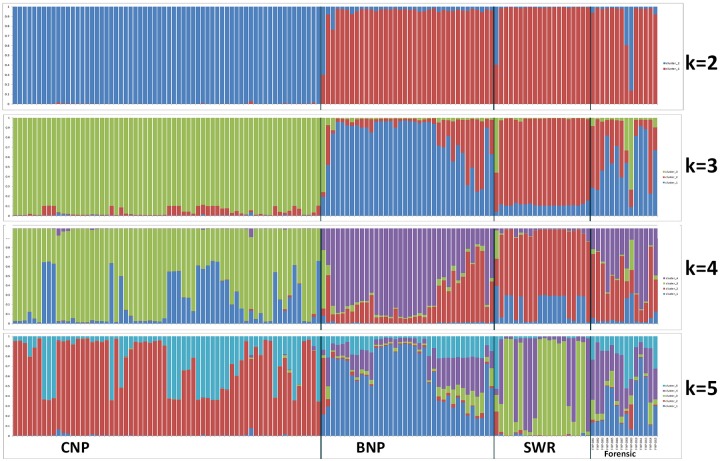
STRUCTURE bar plot of (*K* = 2 to 5) with LOCPRIOR information.

Bayesian clustering analysis performed in STRUCTURE (with LOCPRIOR) predicted two as the most probable number of genetic clusters (*K* = 2) based on Evanno methodology, where one group consisted of samples from CNP, while the other constituted of samples from BNP and SWR (Fig 2 and S4 Fig). However, *K* = 3 was the most supported structure from mean likelihood (mean LnP(K) = -2097.47 and SD = 9.6), and *Q* value plots where the three clusters corresponded to tiger samples from CNP, BNP and SWR. The latter structure pattern with three genetic clusters (*K* = 3) was also corroborated based on *Q*-score plots across CNP, BNP and SWR, making an effective reference structure that would be essential for forensic assignment of unknown samples.

**Fig 3:**
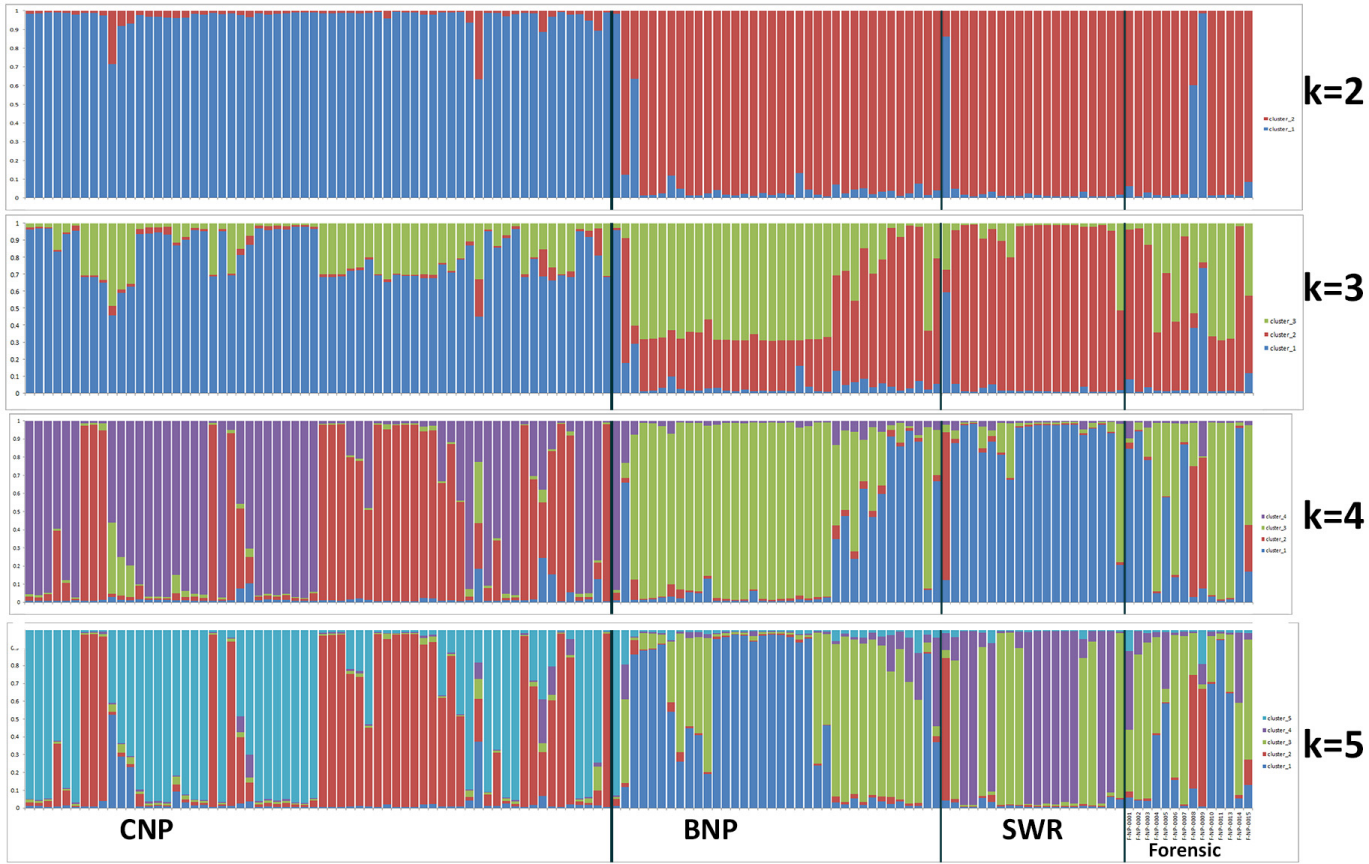
STRUCTURE bar plot of (*K* = 2 to 5) without LOCPRIOR information.

The unsupervised approach in STRUCTURE also predicted two as the most probable number of genetic clusters (*K* = 2) based on Evanno method dividing the data into one cluster consisting of CNP samples and another with samples originating from BNP and SWR (Fig 3 and S5 Fig). However, this model seemed to support *K* = 4 from mean likelihood (mean LnP(*K*) = -1999.7 and SD = 0.34). The CNP population showed two sub-structures at *K* = 4, but no significant spatial patterns were found, rather the samples were panmictic across the CNP site. Therefore, we usedthe supervised model (with LOCPRIOR) with *K* = 3 subpopulation clusters for the population assignment of unknown forensic samples.

### Forensic assignment of unknown samples

**Fig 4:**
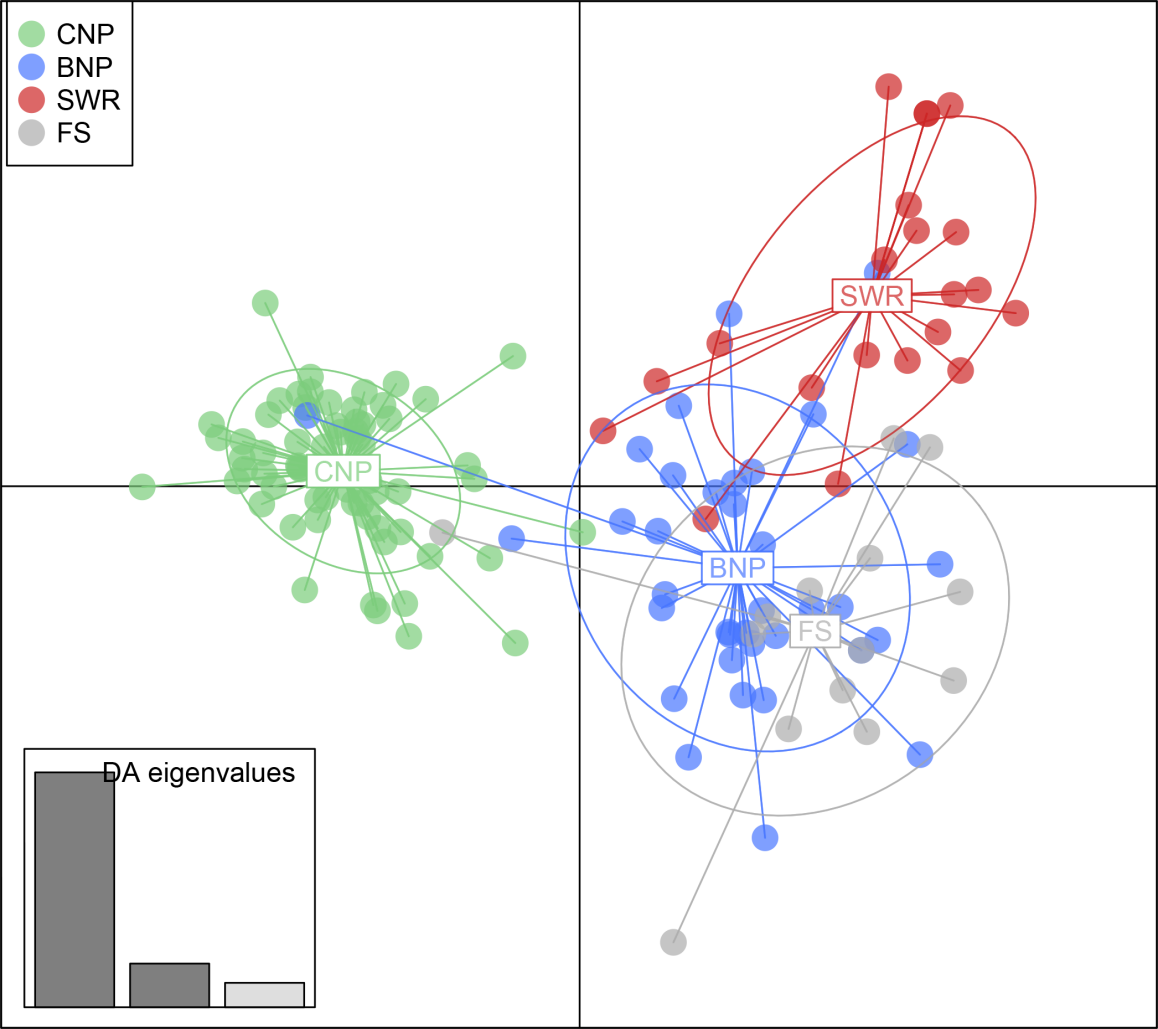
Discriminant analysis of principal components (DAPC) to infer genetic assignment of confiscated tiger parts to source populations in Nepal. Scatter plots of tiger genotypes (known and unknown origin) in relation to discriminant functions were generated using R package *adegenet*, version 2.1.1 [61]. Each point represents one tiger individual and ellipses around each cluster represent 95% confidence. The barplot graphs eigen values of the first three principal components in relative magnitude. BNP, Bardia National Park; CNP, Chitwan National Park; SWR, Shuklaphanta Wildlife Reserve; FS, forensic tiger samples.

Using *K* = 3 as the reference population structure, *Q* membership scores of tiger samples of unknown origin were used to assign them to a geographical location. Eight forensic samples had *Q*-scores above 70% and were assigned to a specific reference population in Nepal, while the remaining six samples were determined to be of admixed genetic makeup (Table 1). Among the eight assigned samples, one sample (F-NP-0009) was assigned to CNP, five samples (F-NP-0004, -0006, -0010, -0011 and -0013) were assigned to BNP, and two samples (F-NP-0002 and -0014) were assigned to SWR. For the admixed individuals, 3 showed greater than 50% ancestry with SWR and most of the remaining ancestry with BNP while the other 3 showed greater than 50% ancestry with BNP and most of remaining ancestry with SWR (2) or CNP (1). Results from Geneclass2 analyses confirmed our findings in STRUCTURE. Using the frequency-based and Bayesian assignment methods, six samples were assigned to BNP, two were assigned to SWR and one was assigned to CNP with a probability >99% (Table 1). As observed in the STRUCTURE analyses, five samples did not meet our assignment probability threshold (>99%) and showed admixed ancestry (Table 1). DAPC analysis suggested that one forensic sample originated from CNP while most others were assigned to the BNP cluster confirming the assignment results obtained using the other methods (Fig 4). Based on our criteria of requiring three or more assignment methods to confirm the results (see methods), we concluded that 6 forensic samples originate from BNP, 2 from SWR and 1 from CNP. 5 samples were classified as unassigned (Table 1).

## Discussion

Wildlife crime is a serious trans-national threat to biodiversity in South Asia/Indian subcontinent and worldwide. There is an immediate need to develop tools that can assist in tracking and ultimately preventing wildlife crime which is driven by organized criminal syndicates that control the burgeoning and highly lucrative illicit trade in wildlife parts[67]. CIB has been actively involved in the investigation of wildlife crime across all regions of Nepal and has had major successes in bringing the perpetrators to justice by closely working with other national and international stakeholders. A high number of seizure cases of tiger body parts from Mid/Far western Nepal compared to other regions indicates that this area might be one of the major trafficking routes for illegal wildlife trade in Nepal. The recently established molecular forensic capability in Nepal provides powerful, new tools in the fight against wildlife crime in Nepal.

The use of molecular forensic tools plays an important role in strengthening law enforcement efforts to address wildlife crime and biodiversity conservation. It has already been successfully used in some countries to improve investigation of wildlife cases, identification of poaching hotspots and trafficking routes for African elephants and Indian leopards, as well as prosecution of wildlife criminals by connecting individuals to crime scenes[20–24, 34, 67].

TAL is a biologically diverse habitat. Once alluvial grasslands and subtropical deciduous forests of TAL has supported the highest recorded density of tigers in the world [68]. Poaching now represents a great threat to tiger survival in this landscape[1]. Dwindling tiger numbers in some protected areas calls for urgent conservation action and requires changes in the current protection strategy for tigers. Tiger populations in the western part of TAL may already have reached a dangerously low tipping-point, and further decline in local population numbers may push the sub-population towards local extinction, similar to what happened to the rhino population across Babai River floodplain in BNP [18, 69].

Application of molecular forensics is relatively new and rapidly evolving, and is gaining acceptance in wildlife conservation efforts[70, 71]. The information obtained through the molecular forensics is more accurate and informative than conventional methods [21, 72]. To identify source population of poached tigers, we created a wild tiger geo-spatial, genetic baseline database through the NTGP[38], which serves as a reference dataset for the forensic analysis. The molecular tracking of tiger parts has provided valuable information on wildlife crime “hot spots” within Nepal and presented scientific evidence to incriminate criminals in a court of law. We identified one tiger skin samples (F-NP-0004; female) that had an exact DNA genotyping profile with one of our female reference NTGP tigers from BNP. We collected scats of that individual in the winter of 2012; this particular individual was probably poached as recently as one to two years ago. Of all the forensic evidence we analyzed, most were traced to BNP (6/15) (Table 1), highlighting this area as a wildlife crime hotspot. This is crucial information for Nepal’s law enforcement officials. After dissemination of these results (Table 1), the concerned stakeholders, including the Nepal Police and the Nepal Army increased their patrols and intelligence activities particularly in the BNP region. As a result, a few of the criminal networks involved in wildlife crime in that area were discovered and necessary actions were taken to prevent further threat from these poachers [73, 74]. Our analysis confirmed that all the seized parts were from tiger and majority of them (10 of 15, >65%) were male.

The molecular assay we have used in our baseline tiger genetic database and forensic samples have high probability of identification (P_ID_; 1.5E-06) and probability of identification among siblings (P_ID (sibs)_; 3.2E-03) with eight microsatellite loci showing high polymorphism [38]. Similarly, our species and sex identification PCR assay was highly effective [44].Other studies have shown that assignment tests can be very effective and accurate when pairwise F_st_ values between source populations exceed 0.05, which was true in our study[44, 65]. Using our analysis framework, we were able to assign 9 of the 14 individuals to a source population in Nepal. Five individuals were unassigned and showed admixed genetic profiles between CNP, SWR and BNP. These individuals may have been offspring of migrants. Previous research detected 3 migrants from SWR to BNP and showed higher gene flow from SWR into BNP than BNP to SWR[44]. This is also reflected in the STRUCTURE *Q* value plots that show a larger proportion of admixed individuals in BNP (Fig 2). Thus, these admixed individuals are more likely to have been poached in BNP than SWR [44]. Forensic samples may also originate from source populations outside of Nepal, which are not represented in our database. To test this hypothesis, we generated Nei’s pairwise genetic distance values for all individuals and built a UPGMA phylogenetic tree to search for outlier samples (S1 File). Two of the unassigned samples (F-NP-0001 and -0008) in this analysis did not cluster with the 3 main groups and may represent individuals from unsampled source populations in India. Overall, the results of our forensic study which used four different analytical methods (STRUCTURE *Q*, GeneClass 2- Assignment and Bayesian likelihood and DAPC) (Table 1) were broadly congruent, indicating that this analysis approach is a highly valuable tool against wildlife crimes in Nepal and beyond. However, our tiger genetic database currently only includes genotypes from tiger populations in Nepal. Hence, we acknowledge that some of the samples assigned to regions in Nepal could have been poached in tiger habitats just across the border in India where tiger allele frequencies are likely similar. There is now an urgent demand to have trans-boundary collaboration between tiger habitat countries [75, 76] with the main goal to build and share standardized tiger genetic data to develop a comprehensive and cross-referencing anti-poaching genetic database. We also recommend a molecular forensic data validation process to evaluate accuracy of the technique and integrate this in courts of law as admissible evidence in wildlife crime cases by conducting multi-year scat surveys and building a robust baseline genetic database of wild tigers not only in Nepal but throughout the tiger range countries in South Asia.

There is an ongoing effort to have court of law in Nepal accept DNA-based evidence for incriminating wildlife criminals. We are working with all the relevant stakeholders, including the United States Fish and Wildlife Service (USFWS) and the International Criminal Police Organization (INTERPOL) to bring awareness on the utility of molecular forensics in Nepal and beyond.

## Conclusions

The world has experienced an unprecedented spike in illegal wildlife trade, threatening to overturn decades of conservation gains. Wildlife trade is the fourth most lucrative illegal trade in the world after drugs, human trafficking and arms trade [77]. To combat wildlife crime through providing scientifically accurate and reliable information, Nepal has now generated a genetic database of wild tiger populations in Nepal, which includes geo-location data that allows molecular forensic tools to determine the source location of seized tiger parts. With efforts like NTGP, developing countries such as Nepal have made significant progress in building local capacity to gather important biodiversity information on various wildlife species. This forensic study provides critical information for effective law enforcement in the fight against wildlife crime.

## Supporting information

S1 Fig Seized tiger skin sample (F-NP-0011) provided by *Central Investigation Bureau*(*CIB*) of Nepal. (DOCX)

S2 Fig A1.5% agarose gel electrophoresis image of tiger species identification PCR targeting cytochrome-b region of the mitochondrial DNA. (DOCX)

S3 Fig A 3% agarose gel electrophoresis image of sex identification PCR of tiger forensic samples targeting Amelogenin gene. Females have asingle band at 214 bp and males havetwo bands at 194 bp and 214 bp.
(DOCX)

S4 Fig Delta *K* plot of STRUCTUREHARVESTER result demonstrating the optimal value (*K* = 3) under LOCPRIOR model. (DOCX)

S5 Fig Delta *K* plot of STRUCTUREHARVESTER result demonstrating the optimal value (*K* = 4) under the model without LOCPRIOR information. (DOCX)

S6 Fig Phylogenetic tree (UPGMA) generated from Nei’s genetic distance using 8 nuclear DNA microsatellite loci for 120 reference tiger samples and 14 forensic samples. Samples are colored based on sampling locations. Green represents CNP, blue represents BNP, and red represents SWR samples. Forensic samples are in black. Clusters or clades are labeled accordingly.
(DOCX)

S1 Table Forensic samples provided by the *Central Investigation Bureau*(*CIB*) of Nepal. (DOCX)

S2 Table Proportions of scat sampled and tigers identified from those scats across each study sites from NTGP. (DOCX)

S3 Table Summary of PCR amplification success, genotyping accuracy and genotyping error rates for 8 microsatellite loci for all processed tiger samples (*n* = 401) collected in three protected areas (Chitwan National Park, Bardia National Park, and Shuklaphanta Wildlife Reserve) to build baseline tiger genetic database. (DOCX)

S4 Table Genotype data on 8 microsatellite loci of the identified tiger forensic samples. (DOCX)

S5 Table Values of mean log-likelihood and Delta K for each assumed *K* (*K* = 1 to 6) of both supervised (with LOCPRIOR) and unsupervised (without LOCPRIOR) models. (DOCX)

S1 File Methodology and results of distance-based phylogenetic analysis. (DOC)

## References

[1] WWF. Overview and Threats of Tiger. Text. http://www.worldwildlife.org/species/tiger. World Wildlife Fund (WWF). 2016.

[2] Perry R. The world of the tiger. Cassell and Comp. Ltd. London 1964. 264 p.

[3] Seidensticker J. Saving wild tigers: a case study in biodiversity loss and challenges to be met for recovery beyond 2010. Integrative zoology. 2010;5(4):285–99. 10.1111/j.1749-4877.2010.00214.x . 21392347

[4] Dinerstein E, Loucks C, Wikramanayake E, Ginsberg J, Sanderson E, Seidensticker J, et al The fate of wild tigers. BioScience. 2007;57(6):508–14.

[5] Barber‐Meyer SM, Jnawali SR, Karki JB, Khanal P, Lohani S, Long B, et al Influence of prey depletion and human disturbance on tiger occupancy in Nepal. Journal of Zoology. 2013;289(1):10–8.

[6] Kenney JS, Smith JL, Starfield AM, McDougal CW. The long‐term effects of tiger poaching on population viability. Conservation Biology 1995 Oct 1;9(5):1127–33. 1995;9(5):1127–33. 10.1046/j.1523-1739.1995.9051116.x-i1 34261258

[7] Gilbert M, Soutyrina SV, Seryodkin IV, Sulikhan N, Uphyrkina OV, Goncharuk M, et al Canine distemper virus as a threat to wild tigers in Russia and across their range. Integrative zoology. 2015;10(4):329–43. 10.1111/1749-4877.12137 25939829

[8] McMurray CA. Testimony of Claudia A. McMurray to the Committee on Natural Resources of U.S. House of Representatives (5 March). Hearing on: poaching American security: impacts of illegal wildlife trade, Washington, D.C., USA. 2008.

[9] Goodrich J, Lynam A, Miquelle D, Wibisono H, Kawanishi K, Pattanavibool A, et al. 2015. Panthera tigris. The IUCN Red List of Threatened Species 2015: e.T15955A50659951. 10.2305/IUCN.UK.2015-2.RLTS.T15955A50659951.en. 2015.

[10] Brown P, Davies B. Trading to extinction. Dewi Lewis Publishing, UK 2014.

[11] Wyler LS, Sheikh PA. International illegal trade in wildlife: Threats and U.S. Policy. Congressional Research Service, USA 2013.

[12] Stoner SS, Pervushina N. Reduced to Skin and Bones Revisited: An Updated Analysis of Tiger Seizures from 12 Tiger Range Countries (2000–2012). TRAFFIC, Kuala Lumpur, Malaysia. 2013.

[13] Yi-Ming L, Zenxiang G, Xinhai L, Sung W, Niemela J. Illegal wildlife trade in the Himalayan region of China.: Kluwer Academic Publishers, Netherlands; 2000. 901–18 p.

[14] Moyle BJ. The black market in China for tiger products. Global Crime 2009;10(1–2):124–43.

[15] Ellis R. Tiger bone & rhino horn: the destruction of wildlife for traditional Chinese medicine. Island Press 2013.

[16] Mainka SA, Mills JA. Wildlife and traditional Chinese medicine: supply and demand for wildlife species. Journal of zoo and wildlife medicine. 1995:193–200.

[17] Gratwicke B, Mills J, Dutton A, Gabriel G, Long B, Seidensticker J, et al Attitudes toward consumption and conservation of tigers in China. PloS one. 2008;3(7):e2544 10.1371/journal.pone.0002544 ; PubMed Central PMCID: PMC2435601. 18596926 PMC2435601

[18] Martin EB, Martin C. Insurgency and poverty: recipe for rhino poaching in Nepal. Pachyderm. 2006;41:61–73.

[19] De Roe. Conservation, crime and communities: case studies of efforts to engage local communities in tackling illegal wildlife trade. IIED, London 2015.

[20] Wasser SK, Mailand C, Booth R, Mutayoba B, Kisamo E, Clark B, et al Using DNA to track the origin of the largest ivory seizure since the 1989 trade ban. Proceedings of the National Academy of Sciences. 2007;104(10):4228–33. 10.1073/pnas.0609714104 PMC1805457 17360505

[21] Alacs EA, Georges A, FitzSimmons NN, Robertson J. DNA detective: a review of molecular approaches to wildlife forensics. Forensic science, medicine, and pathology. 2010;6(3):180–94. 10.1007/s12024-009-9131-7 . 20013321

[22] Budowle B, Van-Daal A. Extracting evidence from forensic DNA analyses: Future molecular biology directions. Biotechniques. 2009;46(5):339 10.2144/000113136 19480629

[23] Wasser SK, Shedlock AM, Comstock K, Ostrander EA, Mutayoba B, Stephens M. Assigning African elephant DNA to geographic region of origin: applications to the ivory trade. Proceedings of the National Academy of Sciences of the United States of America. 2004;101(41):14847–52. 10.1073/pnas.0403170101 ; PubMed Central PMCID: PMC522003. 15459317 PMC522003

[24] Mondol S, Sridhar V, Yadav P, Gubbi S, Ramakrishnan U. Tracing the geographic origin of traded leopard body parts in the indian subcontinent with DNA-based assignment tests. Conservation biology: the journal of the Society for Conservation Biology. 2015;29(2):556–64. 10.1111/cobi.12393 . 25376464

[25] Nagata J, Aramilev VV, Belozor A, Sugimoto T, McCullough DR. Fecal genetic analysis using PCR-RFLP of cytochrome b to identify sympatric carnivores, the tiger Panthera tigris and the leopard Panthera pardus, in far eastern Russia. Conservation Genetics. 2005;6(5):863–6.

[26] Karmacharya DB, Thapa K, Shrestha R, Dhakal M, Janecka JE. Noninvasive genetic population survey of snow leopards (Panthera uncia) in Kangchenjunga conservation area, Shey Phoksundo National Park and surrounding buffer zones of Nepal. BMC research notes. 2011;4(1):516. 22117538 10.1186/1756-0500-4-516 PMC3247909

[27] Gurgul A, Radko A, Słota E. Characteristics of X-and Y-chromosome specific regions of the amelogenin gene and a PCR-based method for sex identification in red deer (Cervus elaphus). Molecular biology reports. 2010;37(6):2915–8. 10.1007/s11033-009-9852-4 19809889

[28] Palomares F, Godoy J, Píriz A, O'Brien S. Faecal genetic analysis to determine the presence and distribution of elusive carnivores: design and feasibility for the Iberian lynx. Molecular ecology. 2002;11(10):2171–82. 12296958 10.1046/j.1365-294x.2002.01608.x

[29] Manel S, Berthier P, Luikart G. Detecting wildlife poaching: identifying the origin of individuals with Bayesian assignment tests and multilocus genotypes. Conservation biology. 2002;16(3):650–9.

[30] Palumbi A, Cipriano F. Species identification using genetic tools: the value of nuclear and mitochondrial gene sequences in whale conservation. Journal of Heredity. 1998;89(5):459–64. 9768497 10.1093/jhered/89.5.459

[31] Baker C, Cipriano F, Palumbi S. Molecular genetic identification of whale and dolphin products from commercial markets in Korea and Japan. Molecular Ecology. 1996;5(5):671–85.

[32] Ogden R, Dawnay N, McEwing R. Wildlife DNA forensics—bridging the gap between conservation genetics and law enforcement. Endangered Species Research. 2009;9(3):175–95.

[33] Lorenzini R. DNA forensics and the poaching of wildlife in Italy: a case study. Forensic science international. 2005;153(2–3):218–21. 10.1016/j.forsciint.2005.04.032 . 15921870

[34] Caniglia R, Fabbri E, Greco C, Galaverni M, Randi E. Forensic DNA against wildlife poaching: identification of a serial wolf killing in Italy. Forensic science international Genetics. 2010;4(5):334–8. 10.1016/j.fsigen.2009.10.012 . 20457032

[35] Wasser S, Brown L, Mailand C, Mondol S, Clark W, Laurie C, et al Genetic assignment of large seizures of elephant ivory reveals Africa’s major poaching hotspots. Science. 2015;349(6243):84–7. 10.1126/science.aaa2457 26089357 PMC5535781

[36] Xu YC, Li B, Li WS, Bai SY, Jin Y, Li XP, et al Individualization of tiger by using microsatellites. Forensic science international. 2005;151(1):45–51. 10.1016/j.forsciint.2004.07.003 15935942

[37] Wan QH, Fang SG. Application of species-specific polymerase chain reaction in the forensic identification of tiger species. Forensic science international. 2003;131(1):75–8. . 12505474 10.1016/s0379-0738(02)00398-5

[38] NTGP/CMDN. Nepal Tiger Genome Project—Final Report 2014, Center for Molecular Dynamics-Nepal. Center for Molecular Dynamics-Nepal, 2014.

[39] PalsbØLl PJ, Berube M, Skaug HJ, Raymakers C. DNA registers of legally obtained wildlife and derived products as means to identify illegal takes. Conservation Biology. 2006;20(4):1284–93. 16922244 10.1111/j.1523-1739.2006.00429.x

[40] Gratwicke B, Seidensticker J, Shrestha M, Vermilye K, Birnbaum M. Evaluating the performance of a decade of Save The Tiger Fund's investments to save the world's last wild tigers. Environmental Conservation. 2007;34(03):255–65.

[41] Bajimaya S, Karki JB, Pant G, Kafle MN, Jnawali SR, Bajracharya S, et al. Tiger Conservation Action Plan for Nepal 2008–2012. Department of National Parks and Wildlife Conservation (DNPWC), Ministry of Forests and Soil Conservation, Government of Nepal. Available: http://www.wwfnepal.org/?191044/Tiger-Conservation-Action-Plan2008-2012. 2007.

[42] Wikramanayake E, McKnight M, Dinerstein E, Joshi A, Gurung B, Smith D. Designing a conservation landscape for tigers in human‐dominated environments. Conservation Biology. 2004;18(3):839–44.

[43] Kanagaraj R, Wiegand T, Kramer‐Schadt S, Anwar M, Goyal SP. Assessing habitat suitability for tiger in the fragmented Terai Arc Landscape of India and Nepal. Ecography 2011 Dec 1;34(6):970–81. 2011;34(6):970–81.

[44] Thapa K, Manandhar S, Bista M, Shakya J, Sah G, Dhakal M, et al Assessment of genetic diversity, population structure, and gene flow of tigers (Panthera tigris tigris) across Nepal's Terai Arc Landscape. PloS one. 2018;13(3):e0193495 10.1371/journal.pone.0193495 29561865 PMC5862458

[45] Dhakal M, Karki M, Jnawali SR, Subedi N, Pradhan NMB, Malla S, et al. Status of Tigers and Prey in Nepal: Department of National Parks and Wildlife Conservation, Kathmandu, Nepal; 2014.

[46] Qiagen. DNeasy Blood & Tissue Handbook 2006. Available from: http://diagnostics1.com/MANUAL/General_Qiagen.pdf.

[47] Bhagavatula J, Singh L. Genotyping faecal samples of Bengal tiger Panthera tigris tigris for population estimation: a pilot study. BMC genetics. 2006;7:48 10.1186/1471-2156-7-48 ; PubMed Central PMCID: PMC1636336. 17044939 PMC1636336

[48] Pilgrim K, McKelvey K, Riddle A, Schwartz M. Felid sex identification based on noninvasive genetic samples. Molecular Ecology Notes. 2005;5(1):60–1.

[49] Janečka J, Jackson R, Yuquang Z, Diqiang L, Munkhtsog B, Buckley‐Beason V, et al Population monitoring of snow leopards using noninvasive collection of scat samples: a pilot study. Animal Conservation. 2008;11(5):401–11.

[50] Menotti-Raymond M, David VA, Lyons LA, Schäffer AA, Tomlin JF, Hutton MK, et al A genetic linkage map of microsatellites in the domestic cat (Felis catus). Genomics. 1999;57(1):9–23. 10.1006/geno.1999.5743 10191079

[51] Sharma R, Stuckas H, Moll K, Khan I, Bhaskar R, Goyal S, et al Fourteen new di‐and tetranucleotide microsatellite loci for the critically endangered Indian tiger (Panthera tigris tigris). Molecular ecology resources. 2008;8(6):1480–2. 10.1111/j.1755-0998.2008.02292.x 21586082

[52] Menotti-Raymond MA, David VA, Wachter LL, Butler JM, O’Brien SJ. An STR forensic typing system for genetic individualization of domestic cat (Felis catus) samples. J Forensic Sci. 2005;50(5):1061–70. 16225210

[53] Gill P, Ivanov PL, Kimpton C, Piercy R, Benson N, Tully G, et al Identification of the remains of the Romanov family by DNA analysis. Nature genetics. 1994;6(2):130–5. 10.1038/ng0294-130 . 8162066

[54] Peakall R, Smouse PE. GENALEX 6: genetic analysis in Excel. Population genetic software for teaching and research. Molecular ecology notes. 2006;6(1):288–95. 10.1093/bioinformatics/bts460 PMC3463245 22820204

[55] Pritchard JK, Stephens M, Donnelly P. Inference of population structure using multilocus genotype data. Genetics. 2000;155(2):945–59. 10835412 10.1093/genetics/155.2.945 PMC1461096

[56] Hubisz MJ, Falush D, Stephens M, Pritchard JK. Inferring weak population structure with the assistance of sample group information. Molecular ecology resources. 2009;9(5):1322–32. 10.1111/j.1755-0998.2009.02591.x 21564903 PMC3518025

[57] Evanno G, Regnaut S, Goudet J. Detecting the number of clusters of individuals using the software STRUCTURE: a simulation study. Molecular ecology. 2005;14(8):2611–20. 10.1111/j.1365-294X.2005.02553.x 15969739

[58] Earl DA. STRUCTURE HARVESTER: a website and program for visualizing STRUCTURE output and implementing the Evanno method. Conservation genetics resources. 2012;4(2):359–61.

[59] Jakobsson M, Rosenberg NA. CLUMPP: a cluster matching and permutation program for dealing with label switching and multimodality in analysis of population structure. Bioinformatics. 2007;23(14):1801–6. 10.1093/bioinformatics/btm233 17485429

[60] Piry S, Alapetite A, Cornuet J-M, Paetkau D, Baudouin L, Estoup A. GENECLASS2: a software for genetic assignment and first-generation migrant detection. Journal of heredity. 2004;95(6):536–9. 10.1093/jhered/esh074 15475402

[61] Jombart T. adegenet: a R package for the multivariate analysis of genetic markers. Bioinformatics. 2008;24(11):1403–5. 10.1093/bioinformatics/btn129 18397895

[62] Wultsch C, Waits LP, Kelly MJ. A Comparative Analysis of Genetic Diversity and Structure in Jaguars (Panthera onca), Pumas (Puma concolor), and Ocelots (Leopardus pardalis) in Fragmented Landscapes of a Critical Mesoamerican Linkage Zone. PloS one. 2016;11(3):e0151043 10.1371/journal.pone.0151043 26974968 PMC4790928

[63] Paetkau D, Calvert W, Stirling I, Strobeck C. Microsatellite analysis of population structure in Canadian polar bears. Molecular ecology. 1995;4(3):347–54. 7663752 10.1111/j.1365-294x.1995.tb00227.x

[64] Rannala B, Mountain JL. Detecting immigration by using multilocus genotypes. Proceedings of the National Academy of Sciences. 1997;94(17):9197–201. 10.1073/pnas.94.17.9197 PMC23111 9256459

[65] Latch EK, Dharmarajan G, Glaubitz JC, Rhodes OE. Relative performance of Bayesian clustering software for inferring population substructure and individual assignment at low levels of population differentiation. Conservation Genetics. 2006;7(2):295–302.

[66] Hauser L, Seamons T, Dauer M, Naish K, Quinn T. An empirical verification of population assignment methods by marking and parentage data: hatchery and wild steelhead (Oncorhynchus mykiss) in Forks Creek, Washington, USA. Molecular Ecology. 2006;15(11):3157–73. 10.1111/j.1365-294X.2006.03017.x 16968262

[67] Iyengar A. Forensic DNA analysis for animal protection and biodiversity conservation: A review. Journal for Nature Conservation. 2014;22(3):195–205.

[68] Sunquist M. Tigers: Ecology and behavior Pp. 19–33 In: Tigers of the World: The science, politics and conservation of Panthera tigris. (eds. Tilson R. & Nyhus P.J.), Elsevier, San Diego, USA 2010.

[69] Thapa K. Situation Analysis of Babai Valley in Bardia National Park- Preliminary Report. WWF Nepal. 2006.

[70] Hsieh HM, Chiang HL, Tsai LC, Lai SY, Huang NE, Linacre A, et al Cytochrome b gene for species identification of the conservation animals. Forensic science international. 2001;122(1):7–18. . 11587860 10.1016/s0379-0738(01)00403-0

[71] Schwartz MK, Luikart G, Waples RS. Genetic monitoring as a promising tool for conservation and management. Trends in ecology & evolution. 2007;22(1):25–33. 16962204 10.1016/j.tree.2006.08.009

[72] Ogden R. Unlocking the potential of genomic technologies for wildlife forensics. Molecular Ecology Resources. 2011;11(s1):109–16. 21429167 10.1111/j.1755-0998.2010.02954.x

[73] Pariyar K. Banjara involvement a new trend in tiger poaching: Police. Republica. 2016 1 13, 2016.

[74] Shahi P. Poaching still a major threat to tigers. Kathmandu Post. 2016 2 12, 2016.

[75] Singh SK, Aspi J, Kvist L, Sharma R, Pandey P, Mishra S, et al Fine-scale population genetic structure of the Bengal tiger (Panthera tigris tigris) in a human-dominated western Terai Arc Landscape, India. PloS one. 2017;12(4):e0174371 10.1371/journal.pone.0174371 28445499 PMC5405937

[76] Gour DS, Reddy PA. Need of transboundary collaborations for tiger survival in Indian subcontinent. Biodiversity and conservation. 2015;24(11):2869–75.

[77] Dalberg W. Fighting illicit wildlife trafficking: A consultation with governments. WWF International, Gland, Switzerland, 2012.

