# Supplementary figures and images for: Species, sex and geo-location identification of seized tiger (Panthera tigris tigris) parts in Nepal—A molecular forensic approach
Source: PLoS One. 2018 Aug 23;13(8):e0201639. doi: 10.1371/journal.pone.0201639 (PMC6107122; doi:10.1371/journal.pone.0201639)

**S1 Fig.** Seized tiger skin sample (F-NP-0011) provided by Central Investigation Bureau(CIB) of Nepal


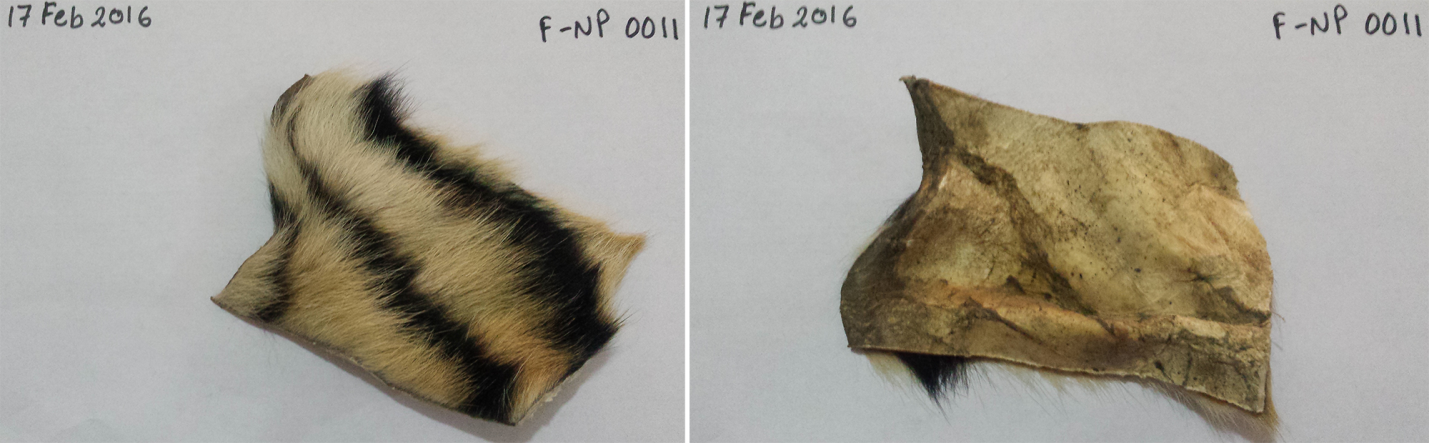

Supplement: S1 Fig — (DOCX) [file pone.0201639.s001.docx]
