# Supplementary material for: Species, sex and geo-location identification of seized tiger (Panthera tigris tigris) parts in Nepal—A molecular forensic approach
Source: PLoS One. 2018 Aug 23;13(8):e0201639. doi: 10.1371/journal.pone.0201639 (PMC6107122; doi:10.1371/journal.pone.0201639)

**S3 Fig.** A 3% agarose gel electrophoresis image of sex identification PCR of tiger forensic samples targeting Amelogenin gene. Females show single band at 214 bp and males show two bands at 194 bp and 214 bp.


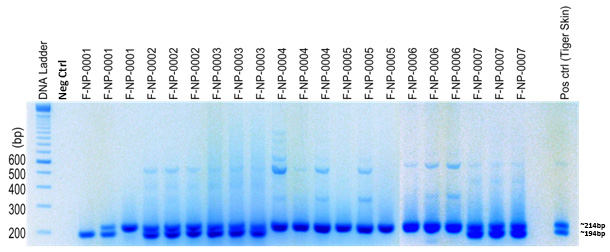

Supplement: S3 Fig — Females have asingle band at 214 bp and males havetwo bands at 194 bp and 214 bp. (DOCX) [file pone.0201639.s003.docx]
