# Supplementary material for: Species, sex and geo-location identification of seized tiger (Panthera tigris tigris) parts in Nepal—A molecular forensic approach
Source: PLoS One. 2018 Aug 23;13(8):e0201639. doi: 10.1371/journal.pone.0201639 (PMC6107122; doi:10.1371/journal.pone.0201639)

**S4 Fig.** Delta K plot of Structure HARVESTER result demonstrating the optimal value (K=3) under LOCPRIOR model.


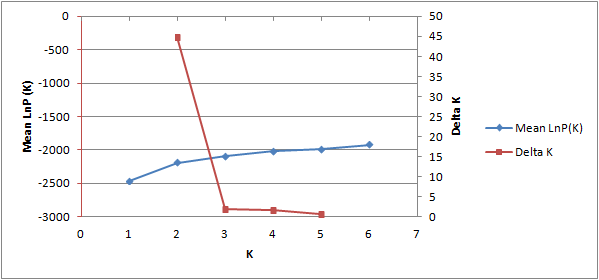

Supplement: S4 Fig — (DOCX) [file pone.0201639.s004.docx]
