# Supplementary material for: Species, sex and geo-location identification of seized tiger (Panthera tigris tigris) parts in Nepal—A molecular forensic approach
Source: PLoS One. 2018 Aug 23;13(8):e0201639. doi: 10.1371/journal.pone.0201639 (PMC6107122; doi:10.1371/journal.pone.0201639)

**S6 Fig.** **Phylogenetic tree (UPGMA) generated from Nei’s genetic distance using 8 nuclear DNA microsatellite loci for 120 reference tiger samples and 14 forensic samples**. Samples are colored based on sampling locations. Green represents CNP, blue represents BNP, and red represents SWR samples. Forensic samples are in black. Clusters or clades are labeled accordingly.


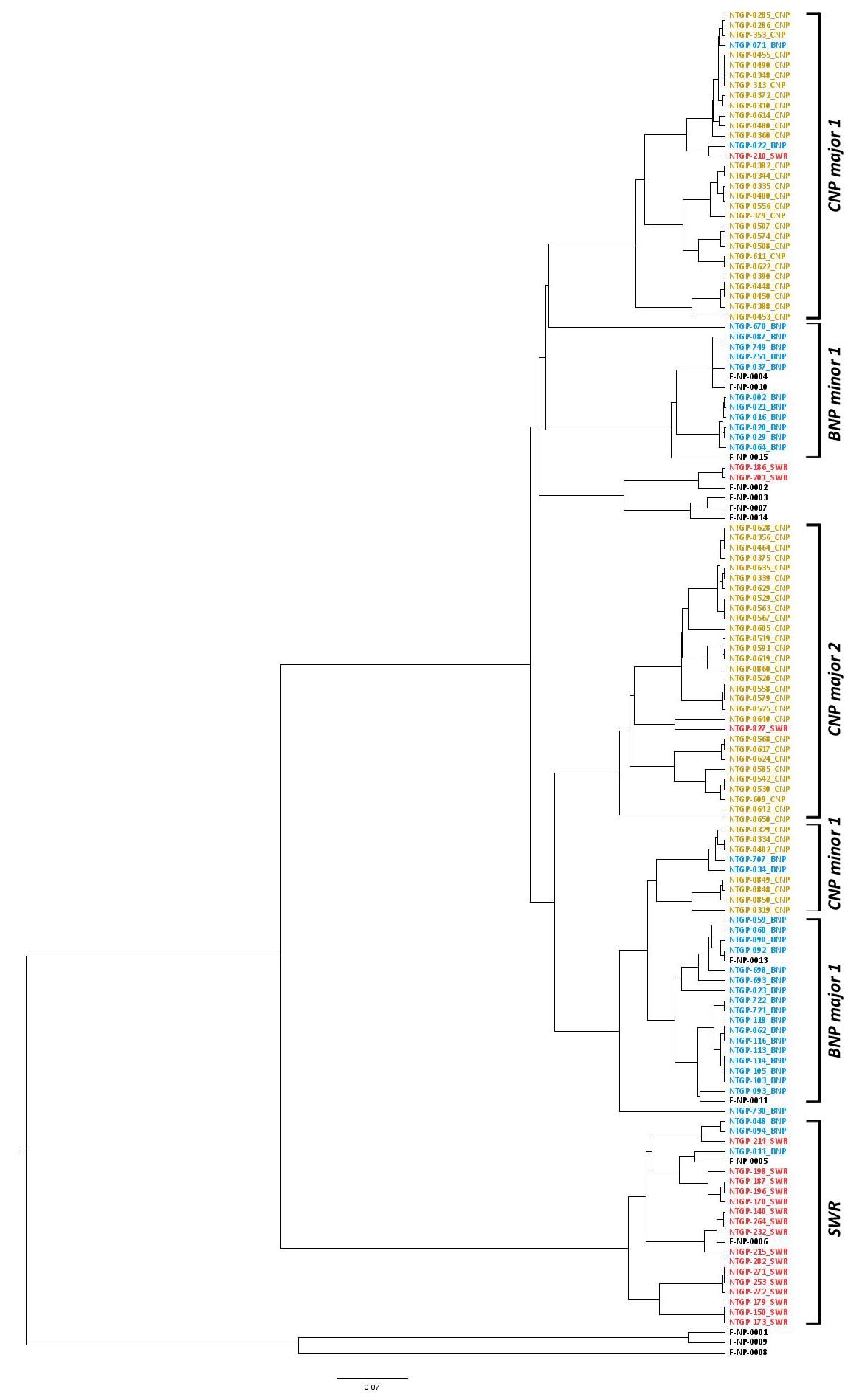

Supplement: S6 Fig — Samples are colored based on sampling locations. Green represents CNP, blue represents BNP, and red represents SWR samples. Forensic samples are in black. Clusters or clades are labeled accordingly. (DOCX) [file pone.0201639.s006.docx]
