# Supplementary material for: Species, sex and geo-location identification of seized tiger (Panthera tigris tigris) parts in Nepal—A molecular forensic approach
Source: PLoS One. 2018 Aug 23;13(8):e0201639. doi: 10.1371/journal.pone.0201639 (PMC6107122; doi:10.1371/journal.pone.0201639)
Supplement: S1 Table — (DOCX) [file pone.0201639.s007.docx]

**S1 Table** Forensic samples provided by the Central Investigation Bureau (CIB) of Nepal

| **Sample** | **Regions of seizure** | **Sample type** | **Year of seizure** |
| --- | --- | --- | --- |
| F-NP-0001 | Far Western Nepal | Skin | 2015 |
| F-NP-0002 | Far Western Nepal | Skin | 2015 |
| F-NP-0003 | Far Western Nepal | Skin | 2015 |
| F-NP-0004 | Mid Western Nepal | Skin | 2015 |
| F-NP-0005 | Mid Western Nepal | Skin | 2015 |
| F-NP-0006 | Mid Western Nepal | Skin | N/A |
| F-NP-0007 | Far Western Nepal | Skin | N/A |
| F-NP-0008 | Eastern Nepal | Skin | 2014 |
| F-NP-0009 | Mid Western Nepal | Skin | 2014 |
| F-NP-0010 | Far Western Nepal | Skin | 2016 |
| F-NP-0011 | Far Western Nepal | Skin | 2016 |
| F-NP-0012 | Far Western Nepal | Blood smeared knife | 2016 |
| F-NP-0013 | Far Western Nepal | Blood smeared knife | 2016 |
| F-NP-0014 | Mid Western Nepal | Skin | 2016 |
| F-NP-0015 | Mid Western Nepal | Skin | 2016 |

N/A= Not available
