# Supplementary material for: Species, sex and geo-location identification of seized tiger (Panthera tigris tigris) parts in Nepal—A molecular forensic approach
Source: PLoS One. 2018 Aug 23;13(8):e0201639. doi: 10.1371/journal.pone.0201639 (PMC6107122; doi:10.1371/journal.pone.0201639)
Supplement: S2 Table — (DOCX) [file pone.0201639.s008.docx]

**S2 Table** Proportions of scat sampled and tigers identified from those scats across each study sites from NTGP.

|  | **CNP** | **BNP** | **SWR** | **Others** | **Total** |
| --- | --- | --- | --- | --- | --- |
| Scat collected | 420 | 116 | 79 | 155 | 770 |
| Tigers identified | 69 | 32 | 19 | 1 | 120 |
