# Supplementary material for: Species, sex and geo-location identification of seized tiger (Panthera tigris tigris) parts in Nepal—A molecular forensic approach
Source: PLoS One. 2018 Aug 23;13(8):e0201639. doi: 10.1371/journal.pone.0201639 (PMC6107122; doi:10.1371/journal.pone.0201639)
Supplement: S3 Table — (DOCX) [file pone.0201639.s009.docx]

**S3 Table** Summary of PCR amplification success, genotyping accuracy and genotyping error rates for 8 microsatellite loci for all processed tiger samples (n = 401) collected in three protected areas (Chitwan National Park, Bardia National Park, and Suklaphanta Wildlife Reserve) to build baseline tiger genetic database.

| **Locus** | **All Samples (*n*=401)** | | | |
| --- | --- | --- | --- | --- |
|  | PCR | GA | ADO | FA |
| *FCA391* | 75.67 | 88.59 | 0.16 | 10.52 |
| *PttD5* | 88.43 | 88.82 | 1.12 | 10.48 |
| *FCA232* | 87.50 | 77.02 | 2.06 | 23.34 |
| *FCA304* | 94.00 | 87.46 | 0.00 | 10.94 |
| *FCA043* | 93.59 | 80.26 | 2.72 | 17.79 |
| *F53* | 65.84 | 84.62 | 0.27 | 13.09 |
| *F85* | 73.86 | 73.42 | 7.94 | 19.01 |
| *FCA441* | 94.85 | 74.73 | 5.40 | 21.69 |
| Mean | 84.22 | 81.87 | 2.46 | 15.86 |
| SD | 10.97 | 6.33 | 2.85 | 5.25 |

PCR, % polymerase chain reaction amplification success; GA, % genotyping accuracy; ADO, % allelic dropout; FA, % false alleles
