# Supplementary material for: Species, sex and geo-location identification of seized tiger (Panthera tigris tigris) parts in Nepal—A molecular forensic approach
Source: PLoS One. 2018 Aug 23;13(8):e0201639. doi: 10.1371/journal.pone.0201639 (PMC6107122; doi:10.1371/journal.pone.0201639)
Supplement: S4 Table — (DOCX) [file pone.0201639.s010.docx]

**S4 Table** Microsatellite (8 loci) profile of the identified tiger forensic samples

| **M. Loci**  **S. ID** | ***FCA391*** | | ***PttD5*** | | ***FCA232*** | | ***FCA304*** | | ***F85*** | | ***FCA043*** | | ***F53*** | | ***FCA441*** | |
| --- | --- | --- | --- | --- | --- | --- | --- | --- | --- | --- | --- | --- | --- | --- | --- | --- |
| F-NP-0001 | 152 | 152 | 199 | 199 | 99 | 101 | 121 | 121 | 161 | 161 | 115 | 115 | 0 | 0 | 111 | 111 |
| F-NP-0002 | 152 | 152 | 199 | 211 | 101 | 101 | 121 | 125 | 157 | 157 | 115 | 115 | 168 | 168 | 115 | 123 |
| F-NP-0003 | 152 | 152 | 199 | 199 | 101 | 101 | 125 | 125 | 165 | 165 | 115 | 119 | 164 | 172 | 115 | 115 |
| F-NP-0004 | 152 | 152 | 207 | 211 | 101 | 101 | 121 | 125 | 165 | 165 | 115 | 115 | 160 | 172 | 115 | 119 |
| F-NP-0005 | 152 | 156 | 199 | 199 | 101 | 101 | 121 | 125 | 157 | 165 | 115 | 117 | 160 | 164 | 119 | 119 |
| F-NP-0006 | 152 | 152 | 211 | 211 | 101 | 101 | 121 | 125 | 165 | 165 | 115 | 123 | 164 | 172 | 111 | 119 |
| F-NP-0007 | 152 | 152 | 199 | 199 | 101 | 101 | 121 | 121 | 165 | 165 | 115 | 115 | 164 | 172 | 115 | 115 |
| F-NP-0008 | 152 | 152 | 199 | 207 | 101 | 101 | 121 | 123 | 153 | 153 | 129 | 129 | 172 | 172 | 107 | 107 |
| F-NP-0009 | 144 | 148 | 199 | 199 | 99 | 99 | 121 | 121 | 0 | 0 | 115 | 115 | 180 | 180 | 111 | 115 |
| F-NP-0010 | 152 | 152 | 207 | 211 | 101 | 101 | 121 | 125 | 165 | 165 | 117 | 123 | 164 | 172 | 119 | 119 |
| F-NP-0011 | 152 | 152 | 207 | 211 | 99 | 101 | 125 | 125 | 157 | 157 | 115 | 115 | 160 | 160 | 107 | 119 |
| F-NP-0013 | 152 | 152 | 207 | 211 | 101 | 101 | 125 | 125 | 0 | 0 | 115 | 115 | 0 | 0 | 107 | 119 |
| F-NP-0014 | 152 | 152 | 199 | 211 | 101 | 101 | 125 | 125 | 161 | 161 | 119 | 119 | 0 | 0 | 115 | 115 |
| F-NP-0015 | 152 | 152 | 207 | 211 | 101 | 101 | 121 | 121 | 165 | 165 | 129 | 129 | 160 | 172 | 107 | 115 |
