# Supplementary material for: Species, sex and geo-location identification of seized tiger (Panthera tigris tigris) parts in Nepal—A molecular forensic approach
Source: PLoS One. 2018 Aug 23;13(8):e0201639. doi: 10.1371/journal.pone.0201639 (PMC6107122; doi:10.1371/journal.pone.0201639)
Supplement: S5 Table — (DOCX) [file pone.0201639.s011.docx]

**S5 Table.** Values of Mean log-likelihood and Delta K for each K (K=1 to 6) for supervised (with LOC PRIOR) and unsupervised (without LOC PRIOR) methods.

| **K** | **with LOC PRIOR** | | **without LOC PRIOR** | |
| --- | --- | --- | --- | --- |
|  | **Mean LnP(K)** | **Delta K** | **Mean LnP(K)** | **Delta K** |
| 1 | -2467.34 |  | -2467.44 |  |
| 2 | -2191.11 | 44.90 | -2191.80 | 2723.10 |
| 3 | -2097.47 | 1.92 | -2097.70 | 1.05 |
| 4 | -2022.26 | 1.67 | -1999.70 | 130.05 |
| 5 | -1993.51 | 0.68 | -1946.75 | 26.50 |
| 6 | -1923.44 |  | -1886.49 |  |
