# Supplementary material for: Species, sex and geo-location identification of seized tiger (Panthera tigris tigris) parts in Nepal—A molecular forensic approach
Source: PLoS One. 2018 Aug 23;13(8):e0201639. doi: 10.1371/journal.pone.0201639 (PMC6107122; doi:10.1371/journal.pone.0201639)
Supplement: S1 File — (DOC) [file pone.0201639.s012.doc]

**S1 File**

**Methodology and Results of Distance-based phylogenetic analysis**

**Phylogenetic inference of microsatellite genotype data**

Nei’s standard genetic distance measure (*Ds*) was utilized to calculate pair-wise genetic distance between samples using their microsatellite genotype data to infer a phylogenetic relationship among samples . A 10,000 bootstrap on loci was performed to construct the distance matrix, which was used to reconstruct topology of phylogenetic tree from UPGMA clustering algorithm . The *Ds* distance matrix calculation and UPGMA tree reconstruction was performed in POPULATIONS 1.2.32 (http://bioinformatics.org/~tryphon/populations) package for Linux. The reconstructed phylogenetic tree was visualized and drawn using FigTree version 1.4.2 (<http://beast.bio.ed.ac.uk/figtree>) software.

**Result of Phylogenetic analysis**

The topology of the phylogenetic tree demonstrated evidence of geo-location based population clusters of the tiger population (S6 Fig). However, some samples collected from one population got assigned into other population cluster. A total of nine samples were assigned into population other than their known collected population site. Four samples that were collected in BNP and two in SWR fell under CNP clades. Three samples from BNP got assigned into SWR clade. The CNP population prominently had two major and one minor clusters, and BNP were grouped into one major and one minor clusters. All CNP clusters were closely placed with two of the BNP clusters, while the SWR cluster formed a very separate group from both CNP and BNP clusters in the tree topology. There were evidences of admixture samples which were placed in clusters of different populations. All forensic samples (n=14) were positioned based on their genetic distance relatedness to individual reference samples in the tree. Based on this, three forensic samples (F-NP-0001 (male), -0008 (female), and -0009 (male)) formed a complete out-group from the tree, which reflected their distant relatedness with our reference tiger genetic database. Among remaining eleven samples, six (F-NP-0004, -0005,-0010, -0011, -0013, and -0015) were closely related to BNP samples while five (F-NP-0002, -0003, -0006, -0007, and -0014) were closely related to SWR samples.

**S6 Fig.Phylogenetic tree (UPGMA) generated from Nei’s genetic distance using 8 nuclear DNA microsatellite loci for 120 reference tiger samples and 14 forensic samples**. Samples are colored based on sampling locations. Green represents CNP, blue represents BNP, and red represents SWR samples. Forensic samples are in black. Clusters or clades are labeled accordingly.

**References**

1. Nei M. Genetic distance between populations. American naturalist. 1972:283-92.

2. Nei M, Tajima F, Tateno Y. Accuracy of estimated phylogenetic trees from molecular data. Journal of Molecular Evolution. 1983;19(2):153-70.

3. Sokol RR, Michener CD. A statistical method for evaluating systematic relationships, Univ. Kansas Science Bulletin. 1958;28:1409-38.
